# A PUFA-rich diet increases endogenous genotoxic stress and mitochondrial DNA damage in mice

**DOI:** 10.1186/s41021-026-00360-4

**Published:** 2026-05-21

**Authors:** Masatomi Shimizu, Ayako Daizo, Kenichi Kawada, Masayo Ikeda, Petr Grúz

**Affiliations:** 1 Division of Medical Nutrition, Faculty of Healthcare, Tokyo Healthcare University, 3-11-3, Setagaya-Ku, Tokyo, 154-8568 Japan; 2 Faculty of Human Nutrition, Seitoku University, 550, Iwase, Matsudo-Shi, Chiba-Ken, 271-0076 Japan; 3 Department of Nutritional Sciences, Faculty of Applied Bio-Science, Tokyo University of Agriculture, 1-1-1, Sakuragaoka, Setagaya-Ku, Tokyo, 156-8502 Japan; 4 Division of Genome Safety Science, Biological Safety Research Center, National Institute of Health Sciences, 3-25-26, Tonomachi, Kawasaki-Ku, Kawasaki-Shi, Kanagawa-Ken, 210-9501 Japan

**Keywords:** Polyunsaturated fatty acids, n-6 fatty acids, Saturated fatty acids, Lipid peroxidation, 1,*N*⁶-etheno-2'-deoxyadenosine, 4-HNE, Mitochondrial DNA damage, Reactive aldehydes, Essential fatty acid deficiency, Mouse dietary model

## Abstract

**Background:**

Substitution of dietary saturated fat with seed oils highly enriched in n-6 polyunsaturated fatty acids (PUFAs) has been advocated as healthy strategy to offset elevated cholesterol levels. However, both n-6 as well as n-3 PUFAs, considered essential because vertebrates lack the enzymatic apparatus for their *de novo* synthesis, are the main source of endogenous DNA damage during the aging process due to their high oxidizability. The membrane pacemaker theory of aging is an extension to the oxidative theory of aging and postulates that higher PUFA content in membrane lipids determines the lifespan of different species.

**Objective:**

We have examined whether a saturated fat-rich diet lacking the essential fatty acids versus a PUFA-rich diet differentially affects lipid profiles and membrane fatty-acid composition, as well as markers of oxidative protein and DNA damage and mitochondrial DNA (mtDNA) integrity in vivo.

**Methods:**

Three-week-old male C57BL/6J mice were fed isocaloric, high-fat diets containing either coconut oil (SFA-rich) or soybean oil (PUFA-rich) for 12 weeks. Plasma and liver lipids were measured, and the fatty acid composition was analyzed in liver and erythrocyte membranes. Endogenous DNA damage was assessed using 1,*N*⁶-etheno-2’-deoxyadenosine (εdA) detection in blood and liver. mtDNA damage and lipid peroxidation derived protein adducts from liver were also examined.

**Results:**

Mice were maintained on a SFA-rich diet for 12 weeks without exhibiting any symptoms of essential fatty acid deficiency (EFAD) as described in historical literature despite the massive synthesis of compensatory n-9 PUFAs. Furthermore, EFAD mice showed reduced levels of endogenous εdA and mtDNA damage as well as protein adducts originating from the primary n-6 PUFA lipid peroxidation product, 4-hydroxy-2-nonenal. However, some other lipid peroxidation-derived protein adducts, such as malondialdehyde and, surprisingly, 4-hydroxy-2-hexenal, were elevated on a SFA-rich diet.

**Conclusions:**

A PUFA-rich diet, relative to the SFA-rich diet, is associated with increased lipid-peroxidation linked adducts and a greater degree of mtDNA damage in vivo, in parallel with membrane enrichment in n-6 PUFA. These findings provide clear evidence of the biological effects of a PUFA-rich diet on endogenous genotoxic stress.

**Supplementary Information:**

The online version contains supplementary material available at 10.1186/s41021-026-00360-4.

## Introduction

The quality of dietary fat plays a crucial role in the development and prevention of lifestyle-related diseases. Traditionally, polyunsaturated fatty acids (PUFAs), particularly n-6 fatty acids like linoleic acid (LA; C18:2 n-6) found in soybean oil, have been recommended for their ability to lower plasma total cholesterol and esterified cholesterol levels [1–4]. These metabolic benefits are widely regarded as protective against cardiovascular diseases [5].

However, the high degree of unsaturation of n-6 PUFAs renders them highly susceptible to lipid peroxidation [6]. This chemical instability leads to the formation of reactive α,β-unsaturated aldehydes, such as 4-hydroxy-2-nonenal (4-HNE), 4-hydroxy-2-hexenal (4-HHE) and malondialdehyde (MDA), which can act as potent genotoxic agents [7]. These molecules can react with DNA to form exocyclic adducts, such as the 1,*N*⁶-etheno-2’-deoxyadenosine (εdA), which are potential initiators of carcinogenesis and established markers of genotoxicity [8]. In other words, a high intake of n-6 PUFAs can alter the lipid composition of cellular membranes, potentially leading to the endogenous accumulation of genotoxic stressors within the intracellular environment. Specifically, 4-HNE has been identified as a key precursor in the formation of mutagenic lesions [9]. While the metabolic advantages of n-6 PUFAs are well-documented, their long-term impact on genome integrity and the aging process remains a critical concern.

In contrast, saturated fatty acids (SFAs), such as those found in coconut oil, have been long criticized for their potential to elevate cholesterol. Although consuming more polyunsaturated PUFA-rich oils can lower total and low-density lipoprotein (LDL) cholesterol levels, it is the PUFAs in LDL that are susceptible to lipid peroxidation. This process causes the lipoprotein and its cholesterol load to be deposited in the atherosclerotic lesions surrounding the arteries [10]. SFAs are completely resistant to lipid peroxidation because they lack reactive carbon-carbon double bonds. Recent research has shed light on the physiological benefits of SFA-induced ketogenesis. Unlike PUFAs, SFAs are resistant to lipid peroxidation and promote the production of ketone bodies, such as β-hydroxybutyrate (β-HB). Crucially, β-HB is no longer viewed merely as an alternative energy source during fasting but as a potent signaling molecule that exerts antioxidant and anti-inflammatory effects [11, 12]. This suggests that SFA consumption transcends mere lipid metabolism, potentially acting as a trigger to actively initiate cellular defense programs via ketone signaling. It has been reported that β-HB can suppress the production of reactive oxygen species (ROS) and enhance the expression of protective genes, potentially safeguarding both nuclear and mitochondrial DNA (mtDNA) from oxidative damage [12–14]. Thus, a SFA-rich diet might offer a unique advantage in maintaining genomic stability through these ketone-mediated defense mechanisms. Therefore, the strategic selection of dietary fatty acids may exert a decisive influence on genomic integrity, which ultimately determines long-term disease susceptibility.

In the present study, we have investigated the trade-off between metabolic profiles and genotoxic risks in mice fed either a PUFA-rich (soybean oil), that contains abundant essential fatty acids, or a SFA-rich (coconut oil), that is essential fatty acids deficient (EFAD), diets. A particular focus was placed on whether SFA-induced ketosis correlates with reduced levels of εdA and improved mtDNA integrity, thereby challenging the conventional paradigm that prioritizes lipid-lowering effects over genomic stability.

## Materials and methods

### Animal experiments

Three-week-old male C57BL/6J mice were purchased from the Jackson Laboratory Japan, Inc. (Yokohama, Japan). All mice were housed in individual cages in a light-controlled environment (lights on 07:00–19:00) with a constant temperature (25 °C ± 2.0 °C) and humidity (50%). They were then randomly assigned to two groups (*n* = 8 per group) and fed ad libitum a purified AIN-93G [15] based high-fat diet containing two types of dietary fat, i.e. SFA (coconut oil) or PUFA (soybean oil) for 12 weeks. 30% of calories were derived from fat. The two groups were designated as SFA group and PUFA group. The diet components included protein 22.1 kcal%, carbohydrate 49.7 kcal% and fat 30.0 kcal% (prepared by Research Diets Inc). The detailed macronutrient composition and fatty acid profiles of the experimental diets are summarized in Tables 1 and 2. Each group of mice were anesthetized with isoflurane and blood was collected by cardiac puncture.

**Table 1:** Ingredient composition of experimental diets

	SFA group diet	PUFA group diet
**Ingredients (g/kg diet)**		
Casein	200.0	200.0
L-Cystine	3.0	3.0
Corn Starch	254.5	254.5
Maltodextrin	132.0	132.0
Sucrose	100.0	100.0
Cellulose	50.0	50.0
**Coconut Oil**	**133.5**	**-**
**Soybean Oil**	**-**	**133.5**
Mineral Mix (S10022G)	35.0	35.0
Vitamin Mix (V10037)	10.0	10.0
Pyridoxine-HCl	0.028	0.028
Choline Bitartrate	2.5	2.5
t-Butylhydroquinone	0.014	0.014
Total (g)	920.542	920.542
Energy (kcal/g)	4.34	4.34
**Macronutrient Composition (kcal%)**		
Protein	20.3	20.3
Fat	30.0	30.0
Carbohydrate	49.7	49.7

SFA group, saturated-fatty-acid-rich diet (coconut oil); PUFA group, polyunsaturated-fatty-acid-rich diet (soybean oil). Diets were formulated based on the AIN-93G purified diet (Research Diets, Inc., Product #D16090602 and D16090603)

Values are presented per kg diet unless otherwise indicated

**Table 2:** Fatty acid composition of experimental diets

Fatty acid	SFA group diet (Coconut oil)	PUFA group diet (Soybean oil)
**Saturated (%)**		
Caprylic acid (C8:0)	7.7	―
Capric acid (C10:0)	5.9	―
Lauric acid (C12:0)	47.6	―
Myristic acid (C14:0)	18.0	0.1
Palmitic acid (C16:0)	8.7	10.4
Stearic acid (C18:0)	10.6	3.9
Total Saturated acid	99.1	15.1
**Monounsaturated (%)**		
Oleic acid (C18:1 n-9)	0.8	23.0
Total Monounsaturated acid	0.8	23.4
**Polyunsaturated (%)**		
Linoleic acid (C18:2 n-6)	0.03	51.8
α-Linolenic acid (C18:3 n-3)	―	7.4
Total Polyunsaturated acid	0.03	59.2
Other fatty acids	0.07	2.3

SFA, saturated fatty acid; PUFA, polyunsaturated fatty acid

Values are expressed as a percentage of total fatty acids. (-) indicate not present in the diet formulation

While fed ad libitum, the mouse groups were monitored for body weight changes and food intake every 3 days/week during the whole experiment. At the end, the liver was collected, weighed and a portion stored in DNA/RNA Shield (Zymo Research, Irvine, USA, Cat.No.R1100) and the remainder was frozen at -80 °C. The blood samples were immediately mixed with EDTA, a portion stored in DNA/RNA Shield (2x concentrate, Zymo Research, Irvine, USA, Cat.No.R1100) and the remainder was subjected to gas chromatography analysis. The plasma was collected for further biochemical analysis.

All studies were approved by the Animal Experimentation Committee of Seitoku University (animal ethical clearance No. 001) and were carried out in accordance with guidelines for the Animal Care and Use Committee of Seitoku University.

### Plasma biochemical analysis and hepatic lipids

Plasma biochemical analysis was performed at the Nagahama Laboratory of Oriental Yeast Co., Ltd. and included: non-esterified fatty acid (NEFA), triacylglycerol (TAG), total cholesterol (T-CHO), cholesterol ester (E-CHO), total ketone bodies (T-KB).

Hepatic lipids were extracted by the method of Folch et al. [16]. TAG and T-CHO concentrations in liver extracts were measured using test kits (LabAssay Triglyceride (Cat.No.291-94501), LabAssay Cholesterol (Cat.No.293-93601) from FUJIFILM Wako Pure Chemical Co., Osaka, Japan).

### Gas chromatography fatty acid analysis

Packed erythrocyte membranes were diluted in 500 µL of water, vortexed, and incubated on ice for 15 min. Lipid extraction was performed according to the method of Barceló-Coblijn et al. [17]. Briefly, 4 mL of isopropanol was added, followed by vigorous vortexing and incubation for 1 h with periodic mixing. Subsequently, 6 mL of hexane was added and mixed for another hour. After centrifugation at 1,200 ×g for 10 min, the upper organic phase containing lipids was collected.

Fatty acids in the liver extracts and erythrocyte membranes were methylated using the boron trifluoride-methanol method [18]. Plasma (0.2 mL) was mixed with 1.5 mL of 0.5 M NaOH in methanol and heated at 100 °C for 9 min. Then, 2 mL of boron trifluoride-methanol complex was added, and the mixture was incubated at 100 °C for 7 min. After cooling, 3 mL of n-hexane and 5 mL of saturated NaCl were added. The mixture was centrifuged at 1,500 × g for 10 min, and the n-hexane phase containing fatty acid methyl esters (FAMEs) was recovered.

FAMEs were analyzed using a Hitachi G-3500 gas chromatograph equipped with a split injector (250 °C) and a flame ionization detector (260 °C) coupled with a D-2500 chromatointegrator. A TC-70 capillary column (60 m × 0.25 mm I.D., 0.2 μm film thickness; GL Sciences, Tokyo) was used with helium as the carrier gas (1 mL/min). The oven temperature program was as follows: 190 °C for 15 min; increased to 200 °C at 5 °C/min and held for 10 min; and finally increased to 210 °C at 5 °C/min and held for 4 min. Peaks were identified by comparison with authentic FAME standards (Funakoshi Co., Tokyo).

For statistical analysis, values below the limit of detection (LOD) were excluded from the calculations. If a specific fatty acid was detected in fewer than 50% of the samples within a group, it was labeled as not detected (ND).

### DNA purification

Total DNA was purified from samples preserved in DNA/RNA Shield using the Quick-DNA MiniPrep Plus Kit (Zymo Research, Irvine, USA, Cat.No.D4068) according to the manufacturer’s instructions. The DNA was eluted in TE buffer (10 mM Tris-HCl, 0.1 mM EDTA, pH 8.0). DNA quantity and purity were determined spectrophotometrically using an Ultrospec 3100 Pro (Amersham Pharmacia Biotech, Uppsala, Sweden).

### Quantification of 1, *N⁶*-etheno-2’-deoxyadenosine 

The levels of εdA in DNA purified from the liver and blood were determined using the OxiSelect™ Aldehyde-Induced DNA Damage ELISA Kit (Cell Biolabs, San Diego, USA, Cat.No.STA-820). The assay was performed in 96-well plates according to the manufacturer’s instructions. The horseradish peroxidase (HRP)-catalyzed chromogenic reaction was measured using a microplate reader (Immuno Mini NJ-2300; InterMed BioTec Co., Tokyo, Japan). The εdA concentrations were quantified by interpolation from a standard curve generated with εdA standards.

### Mitochondrial DNA damage assay

mtDNA damage was assessed using the Mouse DNA Damage Analysis Kit (Detroit R&D, Inc., Detroit, MI, USA, Cat.No.DD2M) according to the manufacturer’s instructions. This assay evaluates mtDNA integrity based on the efficiency with which a long (8.2 kb) mitochondrial target is amplified. In brief, purified liver DNA (50 ng/µL) was subjected to a quantitative polymerase chain reaction (QPCR) reaction to amplify the 8.2 kb fragment. The resulting PCR products were then diluted tenfold with nuclease-free water and quantified by real-time PCR. Threshold cycle values were then used to determine the concentration of the amplified 8.2 kb product by interpolation from a standard curve generated using 8.2 kb DNA standards. A reduction in the amount of the 8.2 kb product was defined as an indicator of increased mtDNA damage.

### Western blot analysis

Liver samples were thawed at room temperature, placed in a bead beater homogenizer (Beads Crusher µT-01; Taitec Co., Saitama, Japan) in 1.0 mL of RIPA Lysis Buffer (ATTO Co., Tokyo, Japan, Cat.No.2332336) solution containing one zirconia balls, and homogenized at 4,600 rpm for 30 s. The resulting homogenates were centrifuged at 14,000 × g for 5 min at 4 °C, and the supernatants were collected. Qubit 2.0 Fluorometer (Life Technologies, USA) was used to measure total protein concentration of each sample. Samples (protein concentration of 2 µg/µL) were diluted with one-part 2 × sample buffer (2 × Laemmli Sample Buffer; Bio-Rad Laboratories, Inc., Cat.No.1610737) and boiled for 5 min. The samples were fractionated in a Criterion TGX stain-free Precast Gel gradient 4% to 15% (Bio-Rad Laboratories, Inc., Cat.No.5678085J10) and transferred to a Trans-Blot^®^ Turbo™ PVDF membrane (Bio-Rad Laboratories, Inc., Cat.No.1704275) using a Trans-Blot Turbo transfer system (Bio-Rad Laboratories, Inc.). Membranes were blocked in EzBlock Chemi (ATTO Co., Tokyo, Japan, Cat.No.2332615) for 60 min, followed by incubation with diluted monoclonal antibodies; each anti-4-HNE (1:1000; clone HNEJ-2, Cat.No.MHH-030n), anti-4-HHE (1:500; clone HHE53, Cat.No.MHH-030n) and anti-MDA (1:1000; clone 1F83, Cat.No.MMD-030n) (all from JalCA, Nikken SEIL Co., Shizuoka, Japan) for 1 h at room temperature. Membranes were then incubated in secondary horseradish peroxidase-goat-anti-mouse antibody solution (diluted 1:7500; Nacalai Tesque, Inc., Kyoto, Japan, Cat.No.21860-74) for 1 h. Bound antibodies were detected using the EzWestLumi Plus reagent (ATTO Co., Tokyo, Japan, Cat.No.2332637) and visualized with a Molecular Imager (Chemi-Doc XRS System; Bio-Rad Laboratories, Inc.). Stain-free gels were also analyzed in the system. Bands were normalized to total protein per lane as described previously [19]. These stain free gels are provided in Supplementary Fig. S1.

### Statistical analysis

Data are expressed as mean ± standard deviation (SD). Outliers were identified and removed using the robust regression and outlier removal (ROUT) method with a coefficient Q = 1%. For comparisons between two groups, the non-parametric Mann-Whitney U test was employed. All statistical analyses were performed using GraphPad Prism version 10.6.0 (GraphPad Software, Boston, MA, USA). A two-sided P-value < 0.05 was considered statistically significant.

## Results

### General health parameters

Table 3 shows the body weight and weight gain of each group assigned to a diet. Although statistical analysis showed no significant difference between the groups, the PUFA group tended to gain more weight than the SFA group (SFA group, 14.23 ± 1.28 g; PUFA group, 16.56 ± 2.47 g). It is also worth noting that total feed intake was almost identical between the two groups (both groups, 231.7 g). Consistent with these results, the PUFA group had significantly higher averages than the SFA group in terms of feed efficiency and retroperitoneal fat, while liver weight increased more in the SFA group than in the PUFA group. The SFA group’s diet is deficient in essential fatty acids. Therefore, deficiency phenotypes such as polydipsia and skin disorders caused by essential fatty acid deficiency are a concern. However, no significant increase in water intake and skin disorders were observed in the SFA group in the present experiment, which involved feeding the SFA (coconut oil) diet for 12 weeks (Table 3 and Supplementary Fig. S1).

**Table 3:** Effects of SFA-rich versus PUFA-rich diets for 12 weeks on body and tissue weight, body weight gain, total feed intake, feed efficiency and water intake

	SFA group	PUFA group
Body weight (g)	25.69 ± 1.64	28.01 ± 3.26
Body weight gain (g)	14.23 ± 1.28	16.56 ± 2.47
Total feed intake (g)	231.7 ± 9.87	231.7 ± 20.13
Feed efficiency (%)	6.14 ± 0.40	7.14 ± 0.50 *
Total Water intake (g)	394.4 ± 101.8	414.9 ± 142.0
Liver weight (g)	1.26 ± 0.04	1.16 ± 0.13 *
Retroperitoneal fat (g)	0.11 ± 0.03	0.17 ± 0.03 *

SFA group, coconut oil diet; PUFA group, soybean oil diet group

Data are expressed as mean ± SD. (*n* = 6–8). *P < 0.05 vs. SFA group

### Lipid profile

To evaluate the metabolic effects of different dietary fatty acid compositions, plasma and liver lipid parameters were compared between the SFA and PUFA diet groups (Table 4). In plasma, T-CHO and E-CHO levels were significantly lower in the PUFA group than in the SFA group, whereas plasma TAG levels were significantly higher. No significant difference was observed in NEFA concentrations. Interestingly, T-KB levels were significantly higher in the SFA group, suggesting increased hepatic ketogenesis despite of adequate saccharide intake. Regarding hepatic tissue, T-CHO and TAG contents did not differ significantly between the groups, although the PUFA group exhibited slightly higher T-CHO and lower TAG levels.

**Table 4:** Effects of dietary fatty acid type on the plasma and liver levels of biochemical markers

	SFA group	PUFA group
T-CHO (mg/dL)	179.4 ± 14.34	146.0 ± 10.69 *
E-CHO (mg/dL)	139.6 ± 12.27	114.8 ± 9.66 *
TAG (mg/dL)	51.00 ± 23.68	90.75 ± 41.43 *
NEFA (µEq/L)	864.3 ± 209.3	767.4 ± 133.6
T-KB (µmol/L)	201.4 ± 51.59	110.8 ± 32.76 *
Liver T-CHO (mg/g liver)	10.42 ± 1.83	12.81 ± 4.64
Liver TAG (mg/g liver)	7.75 ± 2.68	5.97 ± 1.95

SFA group, coconut oil diet; PUFA group, soybean oil diet; T-CHO, total cholesterol; E-CHO, cholesteryl esters; TAG, triacylglycerol; NEFA, non-esterified fatty acids; T-KB, total ketone bodies

Data are expressed as mean ± SD (*n* = 8). *P < 0.05 vs. SFA group

### Distribution of fatty acids in liver and erythrocyte membranes

Table 5 summarizes the fatty acid composition of the liver and erythrocyte membranes in mice fed either the SFA or PUFA diet. The PUFA diet induced pronounced alterations in hepatic fatty acid profiles compared with the SFA diet. In the liver, mice in the PUFA group showed markedly higher levels of stearic acid (C18:0), LA, α-linolenic acid (C18:3 n-3), and arachidonic acid (C20:4 n-6), the latter being a downstream metabolite of LA. In contrast, the PUFA group exhibited significantly lower levels of myristic acid (C14:0), myristoleic acid (C14:1 n-5), palmitoleic acid (C16:1 n-7) and oleic acid (C18:1 n-9). Notably, mead acid (C20:3 n-9) was detectable in the SFA group but absent in the PUFA group. No significant difference was observed in the palmitic acid (C16:0) content between the two groups.

**Table 5:** The fatty acid distribution in liver and erythrocyte membrane

Fatty acids	Distribution of fatty acids (% of total fatty acids)			
	Liver		Erythrocyte membrane	
	SFA group	PUFA group	SFA group	PUFA group
C14:0	0.72 ± 0.34	0.31 ± 0.20 *	ND	ND
C14:1 n-5	2.45 ± 0.42	0.56 ± 0.06 *	ND	0.63 ± 0.11
C16:0	22.99 ± 1.60	23.80 ± 2.09	27.77 ± 2.92	23.60 ± 1.67 *
C16:1 n-7	9.46 ± 1.31	5.01 ± 0.74 *	22.51 ± 6.22	15.75 ± 3.39
C18:0	4.29 ± 0.64	9.99 ± 1.91 *	8.65 ± 2.40	10.79 ± 0.75
C18:1 n-9	48.53 ± 3.37	20.23 ± 2.33 *	23.81 ± 2.62	11.63 ± 0.29 *
C18:2 n-6	1.82 ± 0.21	25.10 ± 1.93 *	ND	19.25 ± 2.34
C18:3 n-3	0.14 ± 0.02	1.50 ± 0.26 *	ND	0.73 ± 0.14
C20:3 n-9	2.75 ± 0.42	ND	6.71 ± 1.85	ND
C20:4 n-6	2.03 ± 0.33	10.41 ± 1.95 *	8.51 ± 2.06	13.68 ± 1.87 *
Other fatty acids	4.84 ± 0.40	2.46 ± 2.13 *	0.45 ± 0.71	2.87 ± 0.90 *
C20:3 / C20:4	1.34 ± 0.02	―	0.80 ± 0.16	―
SFA ratio	28.00 ± 2.02	34.99 ± 3.47 *	36.43 ± 2.37	34.45 ± 1.65
PUFA ratio	6.72 ± 0.90	36.82 ± 2.66 *	16.44 ± 3.76	33.66 ± 3.54 *
MUFA ratio	60.45 ± 2.05	2.05 ± 2.77 *	46.69 ± 4.80	28.18 ± 3.31 *

Fatty acids are shown as carbon number: double bond number and n-x family (e.g., C18:2 n-6)

The C20:3/C20:4 ratio corresponds to the triene/tetraene (T:T) ratio

SFA, coconut oil diet group; PUFA, soybean oil diet group; MUFA, monounsaturated fatty acids; ND, not detected

Data are expressed as mean ± SD.  (liver, n=7-8; erythrocyte membrane, n=5-8). *P < 0.05 vs. SFA group. (-), not calculable

A similar pattern was observed in erythrocyte membranes. The PUFA diet resulted in significantly higher levels of arachidonic acid. In contrast, the PUFA group exhibited significantly lower levels of palmitic acid (C16:0) and oleic acid (C18:1 n-9). Additionally, linoleic and α-linolenic acid (C18:3 n-3) were detected only in the PUFA group, whereas mead acid (C20:3 n-9) appeared exclusively in the SFA group. No significant differences were observed in palmitoleic acid (C16:1 n-7) or stearic acid (C18:0) levels in erythrocyte membranes between the two groups.

These patterns indicate a shift toward monounsaturated fatty acids (MUFAs) in the SFA group and a relative enrichment of n-6 PUFAs in the PUFA group. Mead acid (20:3 n-9), the hallmark of essential fatty acid deficiency (EFAD), appeared in both tissues when the mice were fed an SFA diet [20]. Additionally, Holman originally proposed the mead acid to arachidonic acid ratio (C20:3 n-9 / C20:4 n-6), i.e., the triene / tetraene ratio (T: T ratio), as a quantitative index of essential fatty acid deficiency (EFAD), with a classic threshold around 0.4 indicating deficiency. In subsequent clinical practice and reviews, values ≥ 0.2 are often considered suggestive of EFAD, whereas values ≥ 0.4 are more consistent with overt deficiency. In our data, the ratio was elevated in the SFA group (liver, 1.34 ± 0.02; erythrocyte membranes, 0.80 ± 0.16), whereas it could not be calculated in the PUFA group because mead acid (C20:3 n-9) was undetectable. Thus, these findings are consistent with a biochemical pattern of EFAD in the SFA group, as reflected by the elevated T: T ratio.

### The effects of dietary fatty acids on DNA damage

**Fig. 1:**
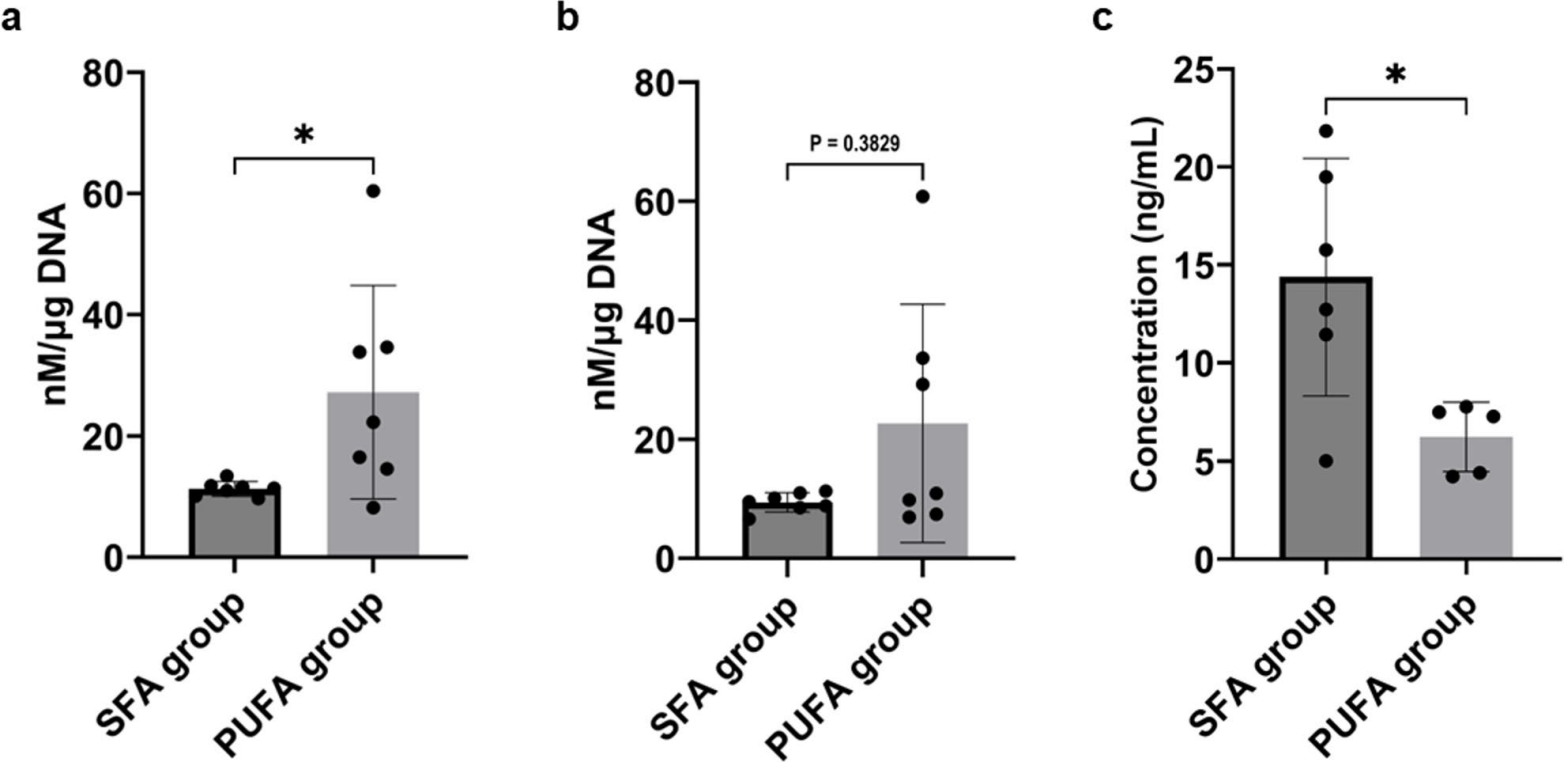
The effects of dietary fatty acids on systemic and liver oxidative DNA damage. Quantification of 1, *N⁶*-etheno-2’-deoxyadenosine (εdA) adducts in the blood (**a**) and liver (**b**). Assessment of mitochondrial DNA (mtDNA) damage in the liver (**c**). εdA is a DNA adduct resulting from lipid peroxidation. The quantity of the 8.2 kb mtDNA product is inversely correlated with the degree of mtDNA damage (i.e., a higher value indicates less damage). Mice were fed high-fat diets based on saturated fatty acids (SFA; coconut oil) or polyunsaturated fatty acids (PUFA; soybean oil). Data are expressed as the mean ± SD (n = 7–8 for εdA; n = 5–6 for mtDNA). Each dot represents an individual mouse. Outliers were identified and removed using the ROUT method (Q = 1%). *P < 0.05 vs. SFA group

To evaluate systemic oxidative stress and DNA integrity, we measured εdA adducts and mtDNA damage (Fig. 1). The PUFA diet significantly increased markers of systemic lipid peroxidation. Specifically, blood εdA, a well-established DNA adduct resulting from lipid peroxidation products (e.g. 4-HNE), was significantly higher in the PUFA group than in the SFA group (Fig. 1a). Liver εdA levels showed a similar trend towards higher values in the PUFA group, albeit non-significant (Fig. 1b). Furthermore, a significant difference was observed in the liver’s susceptibility to mitochondrial damage. The quantity of the long fragment (8.2 kb) of liver mtDNA, which serves as an inverse marker of mtDNA damage (i.e. lower values indicate greater damage), was significantly lower in the PUFA group than in the SFA group (Fig. 1c). These data suggest that a PUFA-rich diet causes a greater degree of liver mtDNA damage than a SFA-rich diet.

Collectively, these data indicate that a PUFA-rich diet is associated with increased lipid peroxidation and a greater degree of hepatic mtDNA damage compared with a SFA-rich diet. These patterns are consistent with the fatty acid profiles in Table 5, wherein the PUFA-rich diet increased liver and erythrocyte n-6 PUFAs (e.g., LA and AA) with concomitant reductions in MUFAs, a composition that plausibly heightens susceptibility to lipid peroxidation and mtDNA damage.

### The effects of dietary fatty acids on liver carbonyl stress

**Fig. 2:**
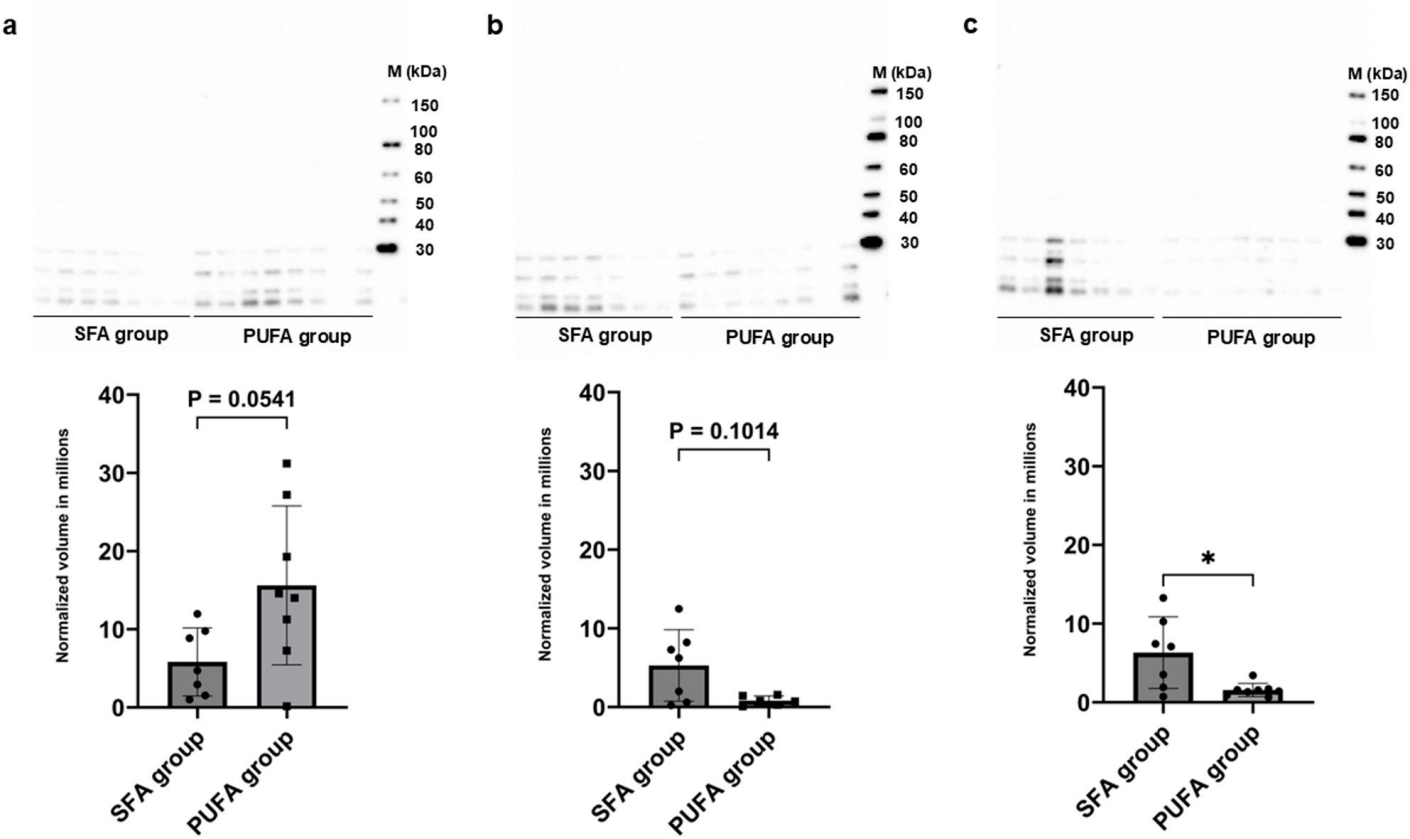
The effects of dietary fatty acids on liver carbonyl stress markers. (**a**) Representative Western blot images and quantification of 4-hydroxy-2-nonenal (4-HNE)-modified proteins in the liver. (**b**) Levels of malondialdehyde (MDA)-modified proteins; a trend toward a difference between the SFA and PUFA groups was observed. (**c**) Levels of 4-hydroxy-2-hexenal (4-HHE)-modified proteins, which were significantly lower in the PUFA group compared with the SFA group. Total protein staining was used for normalization to ensure equal loading. Corresponding stain-free gels are shown in Supplementary Fig. S2. Data are expressed as mean ± SD (n = 7–8 per group). Each dot represents an individual mouse. *P < 0.05 vs. SFA group

To further evaluate the impact of different dietary lipids on oxidative damage, we used Western blot analysis to measure several carbonyl stress markers in the liver (Fig. 2). The total protein content was comparable between the groups, as confirmed by the general protein stain (Fig. 2a). We then assessed the levels of protein-bound lipid peroxidation by-products, specifically 4-HNE. This is a major, highly reactive aldehyde that is derived from n-6 PUFAs, such as LA. There was a trend towards higher levels in the PUFA group than in the SFA group. Based on normalized volume quantification (Fig. 2a), the PUFA group exhibited a higher mean value than the SFA group, though this difference was not statistically significant (Fig. 2a, *P* = 0.054). The levels of protein-bound MDA, another key lipid peroxidation product, also differed between the groups. Quantification revealed a tendency for lower MDA levels in the PUFA group than in the SFA group, though this difference was not statistically significant (Fig. 2b, *P* = 0.101). Interestingly, protein-bound 4-HHE, which is primarily derived from n-3 PUFAs (e.g. α-linolenic acid, C18:3 n-3), was significantly lower in the PUFA group than in the SFA group (Fig. 2c). Although α-linolenic acid (C18:3 n-3) was markedly increased in the PUFA group (Table 5), protein-bound 4-HHE levels were lower than in the SFA group, suggesting that n-6 PUFA–driven lipid peroxidation predominated under PUFA-rich conditions. While there was no significant widespread increase in the PUFA group’s overall carbonyl stress profile, the tendency for higher 4-HNE (an n-6 derivative) combined with higher levels of εdA adducts, as shown in Fig. 1, suggests that metabolism of n-6 PUFAs in the soybean oil diet led to formation of specific reactive intermediates that contributed to the observed DNA damage in this group.

These oxidative patterns also align with the fatty acid distributions reported in Table 5, where the PUFA diet markedly increased hepatic and erythrocyte n-6 PUFA levels (e.g., LA and AA), providing a biochemical context for the observed formation of n-6-derived aldehydes and associated DNA damage.

## Discussion

### The critical trade-off: Metabolic benefits versus genotoxicity risk

The present study investigated the long-term effects of isocaloric high-fat diets based primarily on SFAs (coconut oil) or PUFAs (soybean oil) on the metabolic profile, hepatic lipid dynamics, and oxidative stress status in mice. Our findings reveal a critical trade-off: while the PUFA diet induced certain changes considered metabolic favorable (e.g., lower plasma cholesterol levels), it significantly increased markers of genotoxicity compared to the SFA diet. The most striking novelty of this work is the direct demonstration that the PUFA group exhibited significantly higher levels of εdA adducts in the blood (Fig. 1A) and a greater degree of mtDNA damage in the liver (Fig. 1C). This finding is particularly significant given the established paradigm that dietary fat quality impacts oxidative stress [21]. The εdA is a well-established marker of DNA damage resulting from lipid peroxidation-derived aldehydes, notably 4-HNE [22–24]. Specifically, 4-HNE is predominantly derived from the oxidation of n-6 PUFA, such as LA [25, 26]. The highly unsaturated nature of the PUFA diet (rich in LA; see Table 2) renders it highly susceptible to lipid peroxidation [27]. We propose that this enhanced susceptibility, driven by the structural instability of n-6 PUFA conjugated double bonds, leads to the uncontrolled formation of these reactive intermediates that compromise genomic stability in vivo even in young animals. This compelling evidence is crucial as it suggests that the consumption of high-PUFA vegetable oils, particularly those rich in n-6 PUFAs, may pose a long-term risk of genetic toxicity and cellular dysfunction (e.g., mtDNA impairment leading to energetic deficit), despite any perceived short-term metabolic advantages. This observation urges a deeper evaluation of the long-term safety profile of n-6 PUFA-rich vegetable oils in the context of chronic disease development and aging.

It has been described that the evolutionary strategies to increase longevity in animal species is to reduce the polyunsaturation of their structural lipids [28] or resistance of their mitochondrial respiratory chain complexes to lipoxidative stress e.g. by cysteine depletion [29]. Species such as aquatic animals that do utilize the higly unsaturated n-3 PUFAs to sustain membrane fluidity at low temperatures employ powerfull antioxidants such as astaxanthin or the furan fatty acids to protect against lipid peroxidation [30, 31]. In fact, we have not detected any previously described ill effects of the n-6 and n-3 PUFA deficiency in mice despite of massive mead acid synthesis in the SFA group animals. This raises the question of the essentiality of these PUFAs in homeothermic vertebrates including humans that are not capable of their *de novo* synthesis and suggests that their recommended intake may be greatly overestimated.

### Divergent metabolic pathways: Cholesterol, body weight, and ketogenesis

Consistent with classical nutritional studies, the PUFA group showed lower plasma total T-CHO and E-CHO compared to the SFA group (Table 4). However, this apparent benefit was coupled with greater body weight gain, feed efficiency, and retroperitoneal fat accumulation (Table 3), suggesting a higher propensity for energy storage and adiposity. Conversely, the SFA group displayed a distinct metabolic signature characterized by a profound and significant elevation in plasma T-KB (Table 4). This high level of ketonemia, likely driven by the medium-chain fatty acids (MCFAs) in coconut oil, indicates an enhanced state of hepatic β-oxidation. Previous rodent studies have demonstrated that plasma total ketone bodies increase within one hour after MCFAs administration [32]. A review article in humans also reported that MCFAs increase energy expenditure and β-oxidation compared to long-chain fatty acids [33]. We hypothesize that this increased ketogenic flux is mechanistically linked to the observed lowering of genotoxicity markers in the SFA group. Recent literature highlights that the ketone body β-HB acts as a signaling molecule that can inhibit histone deacetylases and function as an endogenous potent antioxidant. A key study in 2017 detailed the mechanism by which β-HB inhibits histone deacetylase (HDAC) activity, thereby promoting the expression of genes involved in resistance to oxidative stress [34]. Diets rich in SFA are known to be associated with higher levels of DNA repair enzymes involved in damaged nucleotide excision repair [35]. Previous research indicates that PUFAs are more likely than SFAs to induce oxidative stress and DNA damage [36, 37]. These findings strongly suggest that a SFA-rich diet may have provided a more effective cellular defense mechanism against oxidative stress and subsequent DNA damage, possibly through ketone production, than a PUFA-rich diet. Excluding PUFA from diet should minimize the exposure of genome to the toxic lipid peroxides since PUFA rich diets have been described to elevate the levels of various lipid peroxide DNA lesions particularly to drastically increase the εDNA adducts in females [38–40]. The elevation of 4-HHE on the diet lacking any source of n-3 PUFAs compared to soybean oil diet, which contains the linolenic acid, is surprising. It is conceivable that the monoclonal antibodies used for detection can crossreact with some other adducts possibly derived from the Mead acid which is abundant in the SFA group or the adduct being detected is not exclusive the product of n-3 or n-6 PUFAs oxidation. Interestingly, the lower level of protein-bound 4-HHE in the PUFA group is not inconsistent with the increased abundance of α-linolenic acid observed in the liver. The formation of lipid peroxidation-derived aldehyde adducts reflects both fatty acid availability and the dominant oxidative pathways and intracellular compartmentalization. Under PUFA-rich conditions, n-6 PUFA-driven lipid peroxidation appears to predominate, resulting in increased 4-HNE formation, whereas 4-HHE generation may be relatively diminished or rapidly detoxified.

In the present study, aldehyde adducted proteins were predominantly detected in the lower molecular weight range, below ~ 30 kDa. The reason for this distribution is unclear, but identifying and characterising these proteins further will be necessary to clarify the underlying mechanisms.

### Hepatic fatty acid dynamics and oxidative defense

Analysis of liver fatty acid composition (Table 5) confirmed the effects of diet. Notably, oleic acid (C18:1 n-9), the most prominent MUFA species in the SFA group, was present at levels nearly 2.4 times higher than those observed in the PUFA group. Biochemical diagnosis of EFAD is performed by the ratio of Mead acid (20:3 n-9) to AA in tissue (T: T ratio). A ratio exceeding 0.4 suggests EFAD [20]. In this study, mead acid (C20:3 n-9), which is produced during essential fatty acid deficiency, was synthesized. The substantial production of n-9 MUFA and PUFA suggests the presence of a robust compensatory mechanism involving enzyme induction in response to essential fatty acid loading. This *de novo* synthesis of n-9 MUFA and PUFA is crucial for maintaining membrane fluidity and may be an adaptive strategy for sustaining other biological functions including eicosanoid signaling [41]. In addition to the n-9 Mead acid production, cholesterol also plays role in membrane adaptation in that its higher levels increase the membrane fluidity. Therefore, the liver may produce larger amounts of cholesterol under the EFAD conditions as another adaptive mechanism what we have also observed together with bigger liver volume. Furthermore, the susceptibility of lipids to peroxidation in tissues is primarily determined by their fatty acid profile, particularly their degree of polyunsaturation and the content of conjugated double bonds. Monounsaturated fatty acids (e.g., oleic acid) exhibit high chemical stability and strong resistance to lipid peroxidation reactions [28]. Analysis of hepatic carbonyl stress corroborates the findings of oxidative damage. While most carbonyl markers did not reach statistical significance, the increasing trend of 4-HNE in the PUFA group numerically supports the generation of major reactive aldehydes from n-6 PUFAs, which are the precursors of the DNA εdA adduct observed at significantly increased level in blood.

## Conclusion

In conclusion, our data suggests that the choice of dietary fat dictates a complex metabolic outcome where the traditionally perceived health benefits of PUFA (lower plasma cholesterol) are counterbalanced by a significant increase in markers of in vivo genotoxicity and mtDNA damage. Conversely, the SFA-rich diet, characterized by enhanced ketogenesis and adaptive MUFAs synthesis, appears to afford greater protection against lipid peroxidation-induced DNA damage. These findings underscore the need for a nuanced evaluation of qualitative dietary fat intake, extending beyond conventional lipid profiling to include assessments of long-term genomic integrity, cellular damage and compensatory defense mechanisms. Future studies are warranted to establish the long-term health consequences of the observed increase in εdA and mtDNA damage in the context of disease pathogenesis, such as carcinogenesis and aging.

## Electronic Supplementary Material

Below is the link to the electronic supplementary material.

Supplementary Material 1: Fig. S1: No overt EFAD-related skin lesions after 12 weeks of SFA-rich diet. (a) Representative images of mice fed the SFA-rich (EFAD-like) diet for 12 weeks. No alopecia, scaliness, dermatitis, or tail abnormalities were observed. (b) Representative images of mice fed the PUFA-rich diet

Supplementary Material 2: Fig. S2: Stain-free total protein images corresponding to Fig. 2a-c

## Data Availability

No datasets were generated or analysed during the current study.

## Abbreviations

AA: Arachidonic acid (C20:4 n-6)
EFAD: Essential fatty acid deficiency
FAME(s): Fatty acid methyl ester(s)
LA: Linoleic acid (C18:2 n-6)
MDA: Malondialdehyde
mtDNA: Mitochondrial DNA
MUFA(s): Monounsaturated fatty acid(s)
NEFA: Nonesterified fatty acids
PCR: Polymerase chain reaction
PUFA(s): Polyunsaturated fatty acid(s)
SFA(s): Saturated fatty acid(s)
T:T ratio: Triene/tetraene ratio (C20:3 n-9 / C20:4 n-6)
TAG: Triacylglycerol
T-CHO: Total cholesterol
E-CHO: Cholesterol ester
T-KB: Total ketone bodies
εdA: 1,*N⁶*-etheno-2’-deoxyadenosine
4-HHE: 4-hydroxy-2-hexenal
4-HNE: 4-hydroxy-2-nonenal
β-HB: β-hydroxybutyrate
