# Supplementary figures and images for: A PUFA-rich diet increases endogenous genotoxic stress and mitochondrial DNA damage in mice
Source: Genes Environ. 2026 May 21;48:12. doi: 10.1186/s41021-026-00360-4 (PMC13244832; doi:10.1186/s41021-026-00360-4)

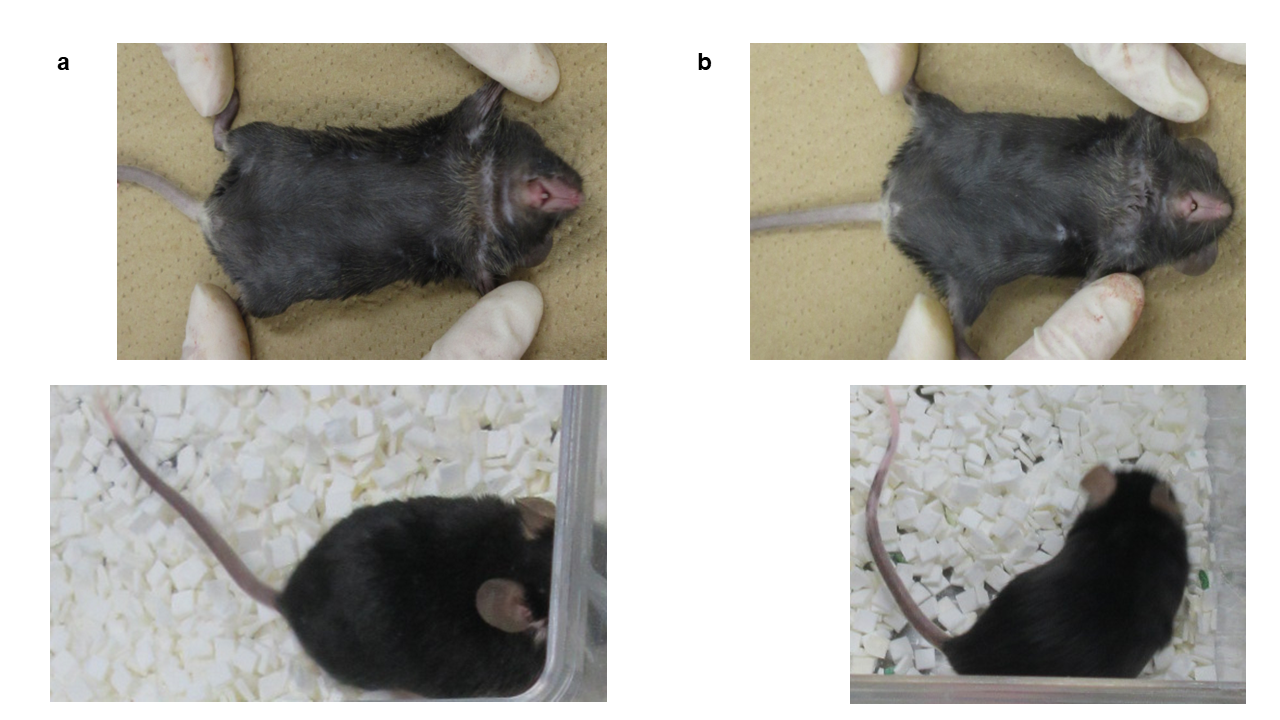

Supplement: Supplementary file 1 — Supplementary Material 1: Fig. S1: No overt EFAD-related skin lesions after 12 weeks of SFA-rich diet. (a) Representative images of mice fed the SFA-rich (EFAD-like) diet for 12 weeks. No alopecia, scaliness, dermatitis, or tail abnormalities were observed. (b) Representative images of mice fed the PUFA-rich diet [file 41021_2026_360_MOESM1_ESM.tif]

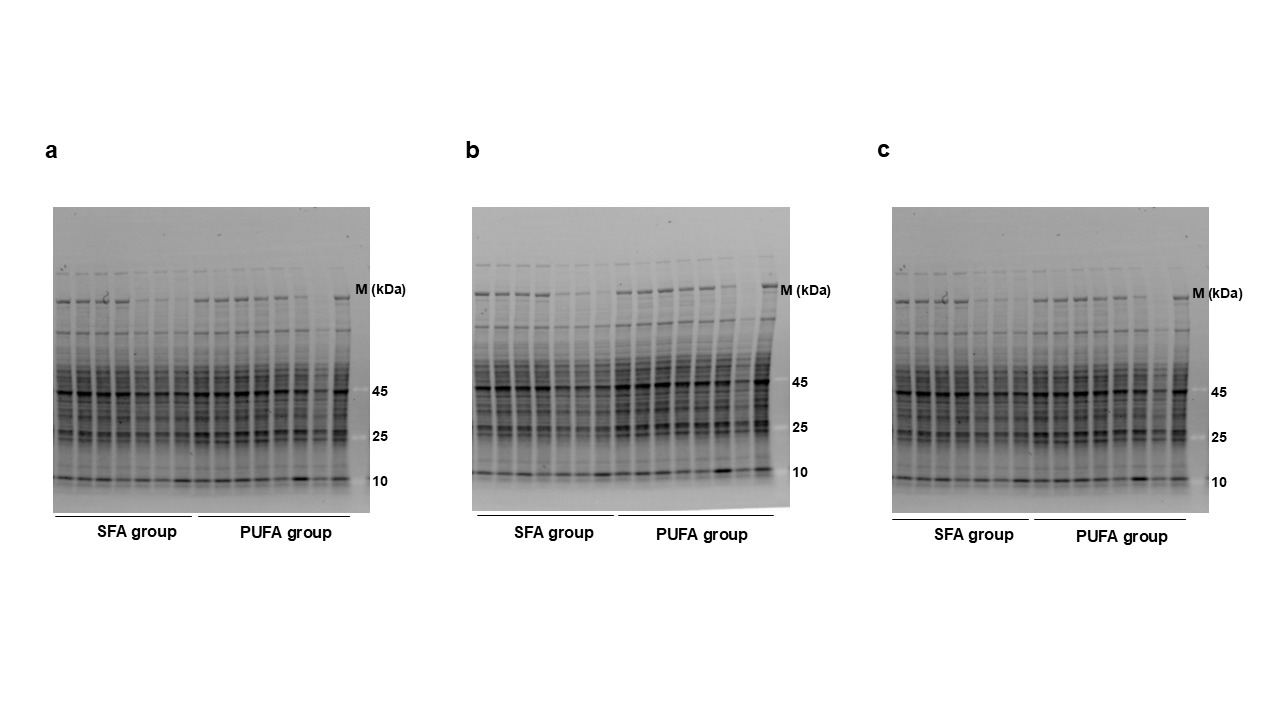

Supplement: Supplementary file 2 — Supplementary Material 2: Fig. S2: Stain-free total protein images corresponding to Fig. 2a-c [file 41021_2026_360_MOESM2_ESM.tif]
